# Large scale integration of drug-target information reveals poly-pharmacological drug action mechanisms in tumor cell line growth inhibition assays

**DOI:** 10.18632/oncotarget.1597

**Published:** 2014-01-21

**Authors:** Richard A. Knight, Mikhail Gostev, Sergei Ilisavskii, Anne E. Willis, Gerry Melino, Alexey V. Antonov

**Affiliations:** ^1^ Medical Research Council Toxicology Unit, Leicester LE1 9HN, UK; ^2^ Molecular Pharmacology Laboratory, Technological University, St-Petersburg, Russia; ^3^ Department of Experimental Medicine & Surgery, University of Rome “Tor Vergata”, 00133 Rome, Italy

## Abstract

Understanding therapeutic mechanisms of drug anticancer cytotoxicity represents a key challenge in preclinical testing. Here we have performed a meta-analysis of publicly available tumor cell line growth inhibition assays (~ 70 assays from 6 independent experimental groups covering ~ 500 000 molecules) with the primary goal of understanding molecular therapeutic mechanisms of cancer cytotoxicity. To implement this we have collected currently available information on protein targets for molecules that were tested in the assays. We used a statistical methodology to identify protein targets overrepresented among molecules exhibiting cancer cytotoxicity with the particular focus of identifying overrepresented patterns consisting of several proteins (i.e. proteins “A” and “B” and “C”). Our analysis demonstrates that targeting individual proteins can result in a significant increase (up to 50-fold) of the observed odds for a molecule to be an efficient inhibitor of tumour cell line growth. However, further insight into potential molecular mechanisms reveals a multi-target mode of action: targeting a pattern of several proteins drastically increases the observed odds (up to 500-fold) for a molecule to be tumour cytotoxic. In contrast, molecules targeting only one protein but not targeting an additional set of proteins tend to be nontoxic. Our findings support a poly-pharmacology drug discovery paradigm, demonstrating that anticancer cytotoxicity is a product, in most cases, of multi-target mode of drug action

## INTRODUCTION

In recent years, the dogma in cancer drug discovery has been the design of selective drugs that target a single protein believed to be critically important for cancer development [1, 2]. Despite an improved understanding of cancer biology, and discovery of multiple genes involved in cancer development and pathology[3-9], the number of successful outcomes for the target-centric approach in anticancer drug design remains disappointingly low[10]. The reason for this could be that cancer pathology involves multiple genes sometimes acting in parallel [11, 12]. From this perspective, poly-pharmacology-based strategies (multi-target drug strategies) represent an attractive alternative [13]. In this case, drugs are specially designed to act on multiple targets (a predefined pattern of proteins) and, therefore, could offer superior efficiency[14]. Although developed to target a specific protein, most currently efficient anticancer drugs are, in fact, essentially poly-pharmacological: they target multiple off-target proteins which might play important, yet unrecognized, roles in the mechanism of action [11, 13-15].

Considering the positive and negative features of poly-pharmacology paradigm, there has been no systematic exploration of whether molecules exhibiting anticancer cytotoxic activity act, as a general rule, in single or multi target mode. To answer this question we undertook a meta-analysis of currently available tumor cell line growth inhibition assays[16]. In total we examined about 70 assays generated by several independent groups (see Table 1) covering in total approximately 500 000 unique molecules. By examining PubChem BioAssay[16] and DrugBank[17] data repositories, we were able to collect experimentally detected protein targets for these molecules. Up to 200 000 molecules have at least one target, either known from the literature or found to inhibit/activate the protein in target oriented screening assays. More than 50 000 molecules were known to target at least 5 proteins. This large scale molecule-to-target information has been used to explore potential therapeutic mechanisms of cancer cytotoxicity in tumor growth inhibition assays.

In the first step we look for “one target” models, i.e. for each assay we identified overrepresented targets among cytotoxic molecules. The results, at first glance, would seem to support the “one target centric” drug discovery paradigm: targeting individual proteins significantly increases the observed odds for a molecule to exhibit strong cancer cytotoxicity. For the top protein targets, depending on the assay, odds ratio [18] varies from 10 to 80 (the odds ratio shows the increase of odds for the molecule to be cytotoxic in the assay if the molecule is known to target the protein in comparison to the odds for all other tested molecules). Moreover, for the topmost targets the odds ratios are relatively consistent across all available assays, despite the fact that the assays were generated using different cancer models and relatively independent chemical libraries.

In spite of this seemingly impressive statistical link between tumour cytotoxicity of a molecule and its ability to target a single protein, deeper analysis of the data convincingly proves that anticancer cytotoxicity is a product, in the majority of cases, of multi-target action mechanisms. First we demonstrate that if a molecule targets a pattern of multiple proteins (i.e. protein “A” and “B” and “C”) then the odds for the molecule to be tumour cytotoxic increases up to 500 fold (see Table 5). Second, we demonstrated that the observed enrichment of “one target” models is just a consequence of the protein involvement in several multi-target models. To prove this, for each enriched “one target” model we eliminated from consideration molecules which target a predefined pattern of proteins (i.e. we excluded from consideration molecules which target protein A and either of proteins B, C, D, etc. where protein A is the enriched target of interest and proteins B, C, D are partners of protein “A” in the enriched multi-target patterns). In every such case, molecules targeting only protein “A” and not targeting proteins (B, C, D ..) have not been overrepresented among cytotoxic molecules in the assay (i.e. targeting only protein A alone does not in general increase chances for a molecule to be tumour cytotoxic; to be tumour cytotoxic, it is imperative for the molecule to target along with protein A also either protein B, C, D etc.). This emphasises that multi-target drug action mechanisms mostly explain anticancer cytotoxic activity of the molecules observed in the assays.

## RESULTS

### Tumor cell line Growth Inhibition assays

We collected publicly available tumor cell line growth inhibition assays. The NCI60 anticancer drug screens include 60 different cell lines covering a wide range of cancer types[19]. We also included several alternative assays developed recently[20, 21]. In total, we used assays developed by 6 independent experimental groups (different cell line models and chemical libraries have been used among assays). The data were downloaded from the PubChem BioAssay repository. In each assay, molecules are classified (classification according to the PubChem BioAssay internal model) into two groups, active and inactive. Active molecules are those which efficiently inhibit tumor cell line growth at relevant concentrations[16]. A summary of these assays is presented in Table 1. The last column provides statistics on the number of tested and active molecules with at least one known protein target.

### Large scale integration of small molecules to protein target data from public sources

The PubChem BioAssay repository stores results of biochemical assays (including HTS), both phenotypic and target oriented. Target oriented assays, in simple terms, can be interpreted as a subset of small molecules found to inhibit (in rare cases to activate) the target protein [2]. The abundance of this information from the PubChem BioAssay repository [16] can be integrated on a large scale to derive binding spectra for approximately one million molecules across a panel of several hundred proteins. This information, although incomplete (some molecules were tested across more than 300 proteins while others only across less than a dozen), can be used to identify molecular mechanisms which unite groups of compounds efficiently inhibiting tumor cell line growth.

We also integrated Drug Bank [17] data for known targets as well as known off-targets for the approved and experimental drugs (some of the approved and experimental drugs are often tested in different assays). The diagram in Figure S1 (supplementary material) presents the distribution of the number of known targets for molecules tested in the assays listed in Table S1 (supplementary material). For example, 234 939 molecules have at least one experimentally validated protein target, 145 365 of them have at least two, and so on.

### Single protein therapeutic models

Considering only individual targets leads to the identification of multiple proteins whose inhibition increases by up to 10–20 fold the observed odds for a molecule to exhibit cancer cytotoxicity. The list of top targets for the IGROV1 Ovarian cell line (NCI60, assay ID 101) is presented in Table 3. For example, NCOA3 was ranked as a top target by p-value of enrichment among active molecules: 68 compounds (out of 231 “active” with at least one experimentally validated target) are known to inhibit NCOA3. In contrast, among the “inactive” group only 37 compounds (out of 2004) are known to inhibit NCOA3. Thus, the odds for molecules known to target “NCOA3” to be “active” in the assay is computed as 68/37 = 1.84 while the odds for molecules not targeting “NCOA3” to be “active” in the assay is computed as (231-68)/(2004-37) = 0.08. Thus, the odds ratio is 22.2. The odds ratio is a measure which is commonly used to estimate the influence of various factors between two measured outcomes [18] (in our case the outcomes are whether the compound is cytotoxic or not, and the factor is whether the compound targets the gene product or not). The odds ratio of 22.2 indicates that the observed odds for molecules targeting “NCOA3” to be cancer cytotoxic is 22 times high than for the molecules which are known to target genes other than “NCOA3”. From Table 2 we can see that targeting individual proteins increases the odds for a molecule to be cytotoxic in the IGROV1 Ovarian cell line.

**Table 1:** Summary of cancer cell line inhibition assays used in meta-analysis

Assay group ID	Assay Title	PubChem Assay ID	Compounds in assay: Active (Tested)	Compounds with targets*: Active (Tested)
NCI60	NCI 60 assays	(AID: 1 to 145)	~2000 (~40 000)	~220 (~2 000)
HPDE_C7	Fluorescent HTS Cytotoxicity/Cell viability assay (HPDE-C7K cells)	431	1 068 (61 593)	1 032 (~46 000)
H69AR_Lung	Human H69AR Lung Tumor Cell Growth Inhibition Assay - 86K Screen	598	5142 (80 068)	4 844 (~63 000)
Lacking_Gene	Compounds that Suppress the Growth of Human Colon Tumor Cells Lacking Oncogenic Beta Catenin Expression	818	2052 (136 706)	2 011 (~105 000)
Lacking_Gene	Compounds that Suppress the Growth of Cells with a Deletion of the PTEN Tumor Suppressor	827	1659 (136 779)	1621 (~105 000)
Cancer_Stem_Cells	Inhibitors of Cancer Stem Cells	2717	(3 190) (297 011)	3 100 (~196 000)
Synthetic_lethality	qHTS for induction of synthetic lethality in tumor cells producing 2HG	686971	7 452 (328 241)	7327 (~230 000)

* compounds with at least one experimentally validated protein target

Similar enrichment of individual targets can be seen in all other tumour growth inhibition assays. The top enriched targets for HCT116 cells with a targeted deletion of the PTEN gene are reported in Table 3. We can see that MITF, IDH1 and CFTR are among the top enriched targets with strikingly high observed odds ratios. Results for other assays are reported in supplementary material.

### Multi-target models

Next we analysed multi-target patterns, i.e. pairs and triplets of proteins (which we refer to as poly-pharmacological models). Multi-target pattern, if targeted by a molecule, increases the odds for the molecule to exhibit cancer cytotoxicity in comparison to the odds for single proteins from the pattern. For example, patterns related to “NCOA3” and the IGROV1 Ovarian cell line are presented in Table 4. We can see a significant increase in the odds for molecules targeting pairs of proteins. For example, odds for the pattern “NCOA3 and NR2E3” are 61.5 which is at least 3 times higher than odds for each individual protein from the pattern.

Similar improvements in the odds ratios are found in other assays using different cancer models (targeted gene deletion, cancer stem cells, etc.) and employing independent chemical libraries. Table 5 reports the top enriched multi target models in growth inhibition assay for HCT116 cells with a targeted deletion of the PTEN gene. The single top model is related to MITF with an impressive odds ratio of 78.6. However, targeting the pair “MITF and ATXN2” increases the odds ratio to 240. Finally, targeting triplet “MITF and ATXN2 and SMAD3” increases the odds ratio still further to 500; 117 molecules which are known to target all 3 genes have demonstrated anticancer cytotoxicity while only 16 molecules targeting the same pattern have been inactive.

**Table 2:** Top individual targets enriched among molecules producing a cytotoxic effect in the IGROV1 Ovarian cell line

P-value FDR corrected	Target (Gene Symbol)	Odds Ratio	The number of active compounds known to target gene	The total number of active compounds (IC50 < 10^−6^)	The number of inactive compounds known to target gene	The total number of inactive compounds (IC50 >10^−6^)
1.82E-42	NCOA3	**22.2**	68	231	37	2004
2.32E-42	NR5A1	**18.1**	73	231	50	2004
7.76E-42	NR5A2	**17.3**	73	231	52	2004
9.26E-38	NCOA1	**7.3**	57	231	26	2004
3.07E-36	IDH1	**24.9**	88	231	123	2004
8.29E-34	CFTR	**9.4**	86	231	128	2004
1.57E-33	PAX8	**8.7**	71	231	76	2004
1.09E-28	MITF	**11.3**	81	231	137	2004
2.49E-28	NFE2L2	**7.4**	77	231	123	2004

**Table 3:** Top individual targets enriched among molecules producing cytotoxic effects in HCT116 cells with a targeted deletion of the PTEN

P-value FDR corrected	Target (Gene Symbol)	Odds Ratio	The number of active compounds known to target gene	The total number of active compounds (IC50 < 10^−6^)	The number of inactive compounds known to target gene	The total number of inactive compounds (IC50 >10^−6^)
MITF	1.00e-300	**78.6**	456	1621	510	102910
GMNN	1.00e-300	**13.6**	682	1621	5220	102910
IDH1	1.00e-300	**22.1**	476	1621	1901	102910
ATXN2	1.00e-300	**74.2**	421	1621	484	102910
EPAS1	1.00e-300	**46.0**	367	1621	650	102910
CFTR	1.00e-300	**32.7**	336	1621	816	102910
TDP1	1.00e-300	**12.2**	1236	1621	21478	102910
SMAD3	2.45e-286	**24.5**	328	1621	1053	102910
HIF1A	4.75e-261	**40.1**	248	1621	462	102910

### Essential polypharmacology of molecules exhibiting anticancer properties

Here we show that the observed odds ratios related to a single protein are, in most cases, a consequence of essentially multi-target mechanisms of action. To demonstrate this, we applied the following procedure. For each significant “one target” model (referred to as protein “A”) we filtered out from consideration molecules which also target one (or several) proteins from those that form significant polypharmacological patterns with protein “A” (referred to as proteins “B”, “C”, …). The aim of the procedure is to see whether molecules targeting protein “A” but not targeting either of the proteins “B”, “C”, .. retain high odds of cytotoxic predictability.

The top single protein model (“NCOA3”) for IGROV1 Ovarian cell line (Table 3) gives an odds ratio of 22 with 68 active molecules and only 37 inactive molecules targeting “NCOA3”. However, 60 active molecules also target “NR5A2” while only 22 inactive molecules do so. The odds ratio for molecules targeting “NCOA3” but not targeting “NR5A2” drops to 6 (8 active molecules and only 15 inactive molecules). Finally, if we filter out molecules targeting “NFE2L2” then the odds ratio drops further to 1.1 with only 1 active and 11 inactive molecules known to target (“NCOA3”) but not either “NR5A2” or “NFE2L2”. This discloses that molecules targeting only “NCOA3” show no increase in potency to inhibit tumour cell growth.

**Table 4:** Top-enriched multi-target patterns with “NCOA3” among molecules producing cytotoxic effects in the IGROV1 Ovarian cell line

P-value FDR corrected	Target (Gene Symbol)	Odds Ratio	The number of active compounds known to target gene	The total number of active compounds (IC50 < 10^−6^)	The number of inactive compounds known to target gene	The total number of inactive compounds (IC50 >10^−6^)
8.43e-40	NCOA3 and NR5A2	**31.6**	60	231	22	2004
1.43e-39	NCOA3 and NFE2L2	**46.7**	54	231	13	2004
5.40e-34	NCOA3 and CFTR	**32.3**	50	231	17	2004
1.52e-29	NCOA3 and PAX8	**36.9**	42	231	12	2004
6.19e-28	NCOA3 and NR2E3	**61.5**	36	231	6	2004
2.26e-09	NCOA3 and MC4R	**59.7**	13	231	2	2004

**Table 5:** Statistics for MITF-related poly-pharmacological patterns in HCT116 cells with a targeted deletion of the PTEN gene

P-value FDR corrected	Target/pattern (Gene Symbol)	Odds Ratio	The number of active compounds known to target gene/pattern	The total number of active compounds (IC50 < 10^−6^)	The number of inactive compounds known to target gene/pattern	The total number of inactive compounds (IC50 >10^−6^)
< 1.00e-286	MITF	**78.6**	456	1621	510	102910
< 1.00e-286	ATNX2	**74.2**	421	1621	484	102910
< 1.00e-286	SMAD3	**24.5**	328	1621	1053	102910
6.55e-279	MITF and SMAD3	**188.5**	189	1621	72	102910
< 1.00e-300	MITF and ATXN2	**240.1**	247	1621	77	102910
< 1.00e-230	MITF and ATXN2 and SMAD3	**500.2**	117	1621	16	102910

The same is true for all other single protein models in all considered tumour growth inhibition assays. For example, in spite of much more impressive statistics for the MITF model (odds ratio 78, see Table 5) in HCT116 cells with a targeted deletion of the PTEN gene, the same analysis revealed underlying multi-target mechanisms. For example, filtering out molecules which target both MITF and ATXN2 resulted in a drop in odds ratio from 78 to 42 with 209 active and 433 inactive molecules. Next, filtering GMNN and IDH1 resulted in a fall in odds ratio to 20 (67 active and 259 inactive molecules). Finally, repeating the iterative procedure for MAP4K2, SMAD3, CXCR6, EHMT2, KCNJ1, HCRTR1, CTDSP1, CHRM5, NCF1, GSTO1, CASP1, ARRB1, INS, NPSR1 and PTBP1 reduced the final odds ratio to 1.6 with only 3 active and 160 inactive molecules which target MITF and target none of the 18 other targets. Therefore, despite the initial impressive odds ratio for MITF, as a single target, after dissecting those molecules which are known to target one or several proteins from the list of 18 proteins (MITF polypharmacological partners in the assay), we see that the remaining molecules which target only MITF are not overrepresented among molecules that inhibit HCT116 cell growth.

**Table 6:** Polypharmacology of currently approved anticancer drugs

Drug	Indication	Drug Bank Targets	PubChem Off-Target
Sunitinib	renal cell carcinoma	8	~150
Sorafenib	advanced renal cell carcinoma, liver cancer	7	~120
Tamoxifen	breast cancer	2	~70
**Paclitaxel**	lung, ovarian, and breast cancer	2	4

Similar results are observed in the other assays used in our study. We present a similar analysis for assay 2717 (inhibitors of cancer stem cell growth) and assay 431 (cell viability assay with HPDE-C7K cells) in supplementary material. In the first case, we considered EPAS1 as a single target and provided similar statistics for multi-target patterns with EPAS1 (supplementary material, Tables S2 and S3) as described above for MITF. Similar, molecules targeting EPAS1 but not targeting 5 other genes tend to be nontoxic (supplementary material, Table S4). The same analysis was performed for IDH1 gene with the similar outcome (supplementary material, Tables S5, S6 and S7).

### Polypharmacology of currently approved anticancer drugs

Although many of the currently approved anticancer agents were designed within a single target oriented approach they, in fact, target multiple proteins. We summarise data for several anticancer agents in Table 6. As can be seen, most anticancer agents inhibit the activity of hundreds of proteins. In these cases, the efficacy of the drugs could be attributed to the fact that all of them target multiple proteins with very diverse functions. Thus, even being designed to affect a single target, the efficiency of the agent is a consequence of a cryptic multi-target action mechanism. Indeed, the targeted poly-pharmacology pattern has not even been presumed to be targeted.

For example, Sunitinib is an oral, small-molecule, multi-targeted receptor tyrosine kinase inhibitor that was approved initially by the FDA for the treatment of renal cell carcinoma [22]. Though it was developed as multi-targeted drug, Sunitinib was presumed to target tyrosine kinases specifically [17]. However, as is apparent from PubChem target oriented screens (approved drugs are frequently present in various chemical libraries and, thus, could be screened versus a wide range of targets), Sunitinib efficiently targets more than 150 proteins with a very diverse functional background. Again, Tamoxifen is assumed to be a selective estrogen receptor modulator [23]. Like Sunitinib, Tamoxifen has been tested in multiple target oriented screens and has demonstrated the ability to modulate up to 70 other proteins. This shows clearly that most of the currently approved anticancer agents are, in fact, essentially poly-pharmocological.

## DISCUSSION

Multi-target based strategies are attracting increased attention as an alternative approach to discover potent anticancer agents. In contrast to the one target centred approach, which concentrates on promiscuous drugs inhibiting/activating exclusively one protein (or a group of very functionally related proteins), the polypharmocology paradigm is based on multi-target-oriented drugs able to inhibit/activate a predefined pattern of multiple proteins. However, there has been no systematic exploration whether multi-target molecules would demonstrate high efficiency. Here we have undertaken a first large scale analysis of available tumor cell line growth inhibition assays with the primary aim of understanding whether efficient anticancer molecules target a single protein or have multi-target mechanisms of action. Our analysis convincingly proves that inhibition of only one target (if we exclude molecules which also target other proteins) results in no increase of odds for a molecule to be an efficient inhibitor in tumor cell line growth inhibition assays, while inhibition of multi-target patterns of proteins drastically increases the odds for anticancer activity up to 500 fold (see Table 5).

The improved efficiency of multi-target drugs is likely, in part, to result from their ability to prevent cell compensatory mechanisms in response to inhibition of a single protein. Cancer cells can readily switch on alternative pathways, thus reducing the drug efficiency achieved by inhibition/activation of a single protein. The same response in the case of inhibition of multiple proteins would be predicted to be much less efficient. In principle, our approach can be used to identify multi-target protein patterns whose inhibition leads to a high probability that a molecule is cytotoxic for cancer cells. In addition, targeting several targets can cause selective cytotoxicity of the molecule to cancer cells, while sparing normal cells [24, 25].

Finally, we would like to emphasise that although we have collected almost all available information regarding experimentally validated protein targets for small molecules which have been tested in tumor cell line growth inhibition assays, the information is obviously incomplete as only roughly 400 proteins are covered and even for those the information is not systematic (some molecules were tested against several hundred proteins while most against only a few dozen). This only strengthens the conclusion of the paper that, in most cases, anticancer cytotoxicity is a product of multi-target action mechanisms. Because the missing information can only reduce the number of active molecules which target only one protein, we would therefore probably see no molecules at all which can produce anticancer cytotoxic effects by targeting only one protein.

## MATERIALS AND METHODS

### Tumor cell line Growth Inhibition assays

We collected publicly available tumor cell line growth inhibition assays (ftp://ftp.ncbi.nlm.nih.gov/pubchem/Bioassay/). The summary of assays used in our study is presented in Table 1. We used the Pubchem Bioassays model which classifies molecules tested in an anticancer assay into active (producing tumour growth inhibition) and inactive.

### Molecule- to- protein target data: Pubchem Bioassays and Drug Bank

We have integrated two sources of data regarding protein targets of small molecules. Pubchem Bioassays (ftp://ftp.ncbi.nlm.nih.gov/pubchem/Bioassay/) deposits target oriented compound screens (including high throughput and literature derived information) across a wide range of protein targets [16]. Molecules in Pubchem Bioassays target oriented screens are classified as active, inactive and inconclusive. In simple terms, molecules classified as active have demonstrated inhibition/activation of the protein at relevant concentrations. Active molecules are considered here as molecules which target the protein. Drug Bank [17] is a database of currently accepted drug protein targets and drug off targets. The data were downloaded from http://www.drugbank.ca/downloads. Overall statistics for the number of targets for molecules (including data both from DrugBank and Pubchem Bioassays) tested in tumor cell line growth inhibition assays are presented in supplementary material (Table S1 and Figure S1).

### Inference of Therapeutic models of cancer cytotoxicity

To identify single target therapeutic models for each assay we repeated the following procedure [26, 27]. Let us denote KA to be the number of active molecules in the assay and KB to be the number of inactive. For each protein “Z” we calculated two numbers, kA is the number of active molecules which are known target “Z” and kB is the number of inactive molecules which target Z. Odds for the molecules (to be active) targeting “Z” is (kA/kB) while the same odds for the molecules which do not target “Z” is ((KA-kA)/(KB- kB)). The odds ratio is (kA/kB) / ((KA-kA)/(KB- kB)) and indicates the increase/decrease of odds of a molecule to be cytotoxic if the molecule is known to target protein “Z”. The odds ratios for all targets are computed. Significance of the odds ratio is computed using X^2^-distribution and adjustment of p-values for multiple testing (each target is one hypothesis) and is performed by the FDR procedure[28].

Similarly, to identify multi-target therapeutic models, we computed similar statistics (kA and kB) but in this case for all pairs (triplets, etc) of targets. In this case, for each pair of targets “Z1” and “Z2”, kA is the number of active molecules which are known to target both “Z1 and Z2” and kB is the number of inactive molecules which target both “Z1 and Z2”. The number of tested hypothesis is equal, in this case, to all possible pair (triplet, etc) combinations of targets. The p-values are adjusted accordingly [27, 29, 30].

### Disclosure of Potential Conflicts of Interest

No potential conflicts of interest.

## Supplementary Figure


