# Supplementary material for: Large scale integration of drug-target information reveals poly-pharmacological drug action mechanisms in tumor cell line growth inhibition assays
Source: Oncotarget. 2014 Jan 21;5(3):659–66. doi: 10.18632/oncotarget.1597 (PMC3996666; doi:10.18632/oncotarget.1597)
Supplement: Supplementary file 1 [file oncotarget-05-0659-s001.pdf]

Supplementary material for "Large scale integration of drug-target information reveals poly-pharmacological drug action mechanisms in tumor cell line growth inhibition assays"

By examining PubChem BioAssay and DrugBank data repositories, we were able to collect experimentally detected protein targets for these molecules. Up to 200 000 molecules have at least one target, either known from the literature or found to inhibit/activate the protein in target oriented screening assays. More than 50 000 molecules were known to target at least 5 proteins. In total, more than 1500 proteins are covered (at least one molecule is known to target the protein). This large scale molecule-to-target information is next used to explore potential therapeutic mechanisms of tumour cytotoxicity in tumor growth inhibition assays.

Table S1 provides statistics related to the top proteins in terms of the number of molecules known to target them (for molecules tested in the cancer assays from Table 1). In total, 1595 genes are covered (i.e. at least one molecule is known to target one gene product). For 352 genes at least 50 molecules were experimentally found to target its gene product.

Here we provide additional results similar to results presented in the main paper but for other tumor cell line growth inhibition assays. The aim is to demonstrate that nevertheless for each assay the top enriched targets and multi-target enriched patterns varied, the structure of the patterns is the same: odds ratios for individual protein targets are varied from 10 up to 70 folds. Considering pairs of targets increase odds up to 30–150 fold. Considering triplets may result in increase of odds up to 500 folds. Similar, for each enriched single protein target, filtering out molecules that also target multi-target patterns (partners of the protein of interest) results in no observed increase of odds ratio for molecules to be efficient inhibitors of tumor cell line growth.

Inference of Therapeutic models of cancer cytotoxicity

Our goal is to understand the molecular mechanisms of cancer cytotoxicity in terms of protein targets whose inhibition leads to increased chances that the molecule would be cytotoxic. As a first step, we search for single protein targets overrepresented among active molecules (versus inactive molecules). Next we refer to such proteins as "enriched proteins" and the odds ratio is used as a quantitative measure of enrichment12. The odds ratio compares the odds for molecules to be cytotoxic which target the protein of interest with the odds for molecules which do not. Statistical significance is computed using X2 distribution and, next, is adjusted for multiple testing (each target corresponds to one hypothesis, see methods for details).

We also look for patterns of multiple proteins (i.e. protein "A" and "B") which, if being targeted by a molecule, significantly increase the odds (observed in the assay) for the molecule to be cytotoxic in comparison to the odds related to each individual protein from the multi-target pattern. Thus we end up with the patterns consisting of several proteins which mark a subset of molecules (each molecule is known to target each protein from the pattern) with a highly significant proportion of them exhibiting cancer cytotoxicity in the assay. We refer to such patterns as the "poly-pharmacology therapeutic model" of cancer cytotoxicity.

In the tables below:

kA - The number of active molecules known to target gene/pattern

KA - The total number of active molecules ($IC50 < 10^{-6}$)

kB - The number of inactive molecules known to target gene/pattern

KB - The total number of inactive molecules in the assay ($IC50 > 10^{-6}$)

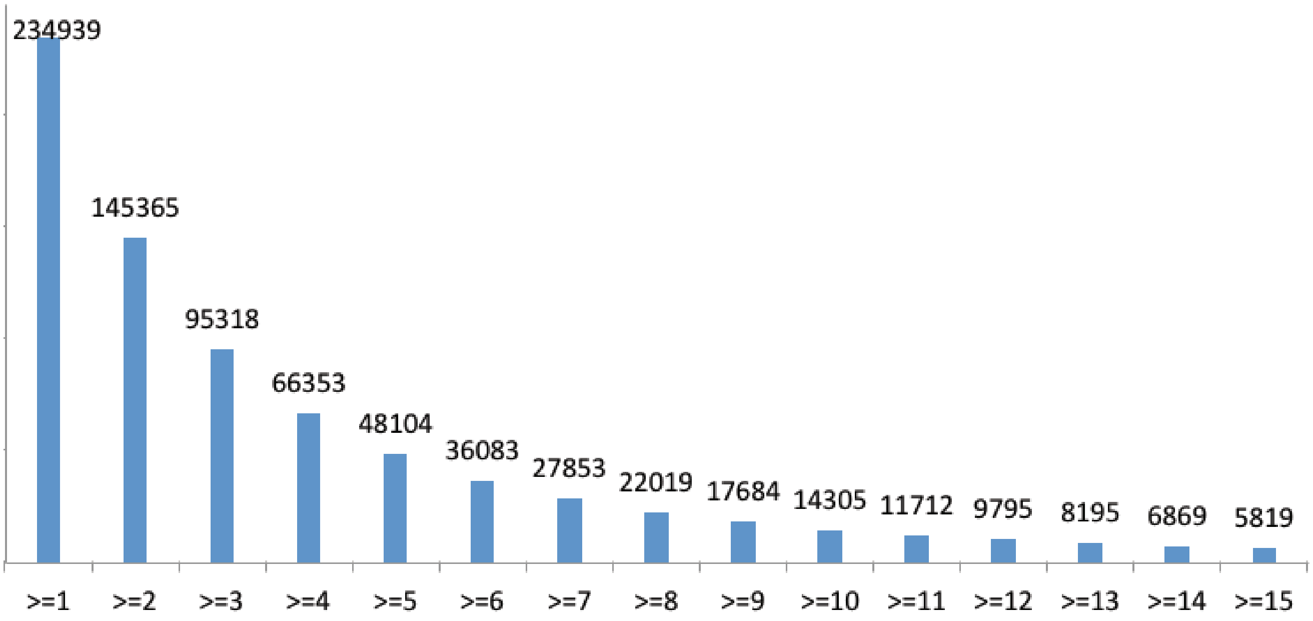

Figure S1: Distribution of the number of experimentally detected targets for ~ 500 000 molecules tested in cancer cell line inhibition assays from Table 1.

Table S1: Top 10 proteins in terms of the number of molecules experimentally validated to target them

Target	The number of molecules
TDP1	58 862
EHMT2	29 085
CYP2C19	20 849
CYP2C9	20 635
ALDH1A1	15 488
BAZ2B	14 399
GMNN	13 360
DRD1	12 831
ATAD5	12 097
DRD3	11 263

Data for "Luminescence Cell-Based Primary HTS to Identify Inhibitors of Cancer Stem Cells"

Table S2: Top individual targets enriched among molecules producing cytotoxic effect in "Luminescence Cell-Based Primary HTS to Identify Inhibitors of Cancer Stem Cells"

#	Target (Gene)	P-value FDR adjusted	Odds Ratio	kA	KA	kB	KB
1	MITF	1.00e-300	27.1	569	3100	1586	192912
2	GMNN	1.00e-300	10.3	1120	3100	9998	192912
3	IDH1	1.00e-300	17.1	803	3100	3865	192912
4	SMAD3	1.00e-300	16.8	527	3100	2325	192912
5	ATXN2	1.00e-300	26.0	525	3100	1500	192912
6	EPAS1	1.00e-300	21.4	440	3100	1480	192912
7	CFTR	1.00e-300	18.7	549	3100	2200	192912
8	TDP1	1.00e-300	7.3	2082	3100	42111	192912
9	PAX8	8.98e-268	17.6	348	3100	1378	192912
10	NOD1	6.94e-247	12.1	390	3100	2262	192912

Table S3: Poly-pharmacology of molecules targeting EPAS1 in "Luminescence Cell-Based Primary HTS to Identify Inhibitors of Cancer Stem Cells"

Target(Gene/ pattern)	P-value FDR adjusted	Odds Ratio	kA	KA	kB	KB
EPAS1	1.00e–300	**21.4**	440	3100	1480	192912
MC4R	1.00e–141	**6.3**	350	3100	3825	192912
CHRM4	4.11e–53	**4.0**	199	3100	3264	192912
EPAS1 and MC4R	2.47e–168	**70.2**	137	3100	127	192912
EPAS1 and CHRM4	2.02e–95	**76.9**	76	3100	63	192912
CHRM4 and EPAS1 and MC4R	1.83e–53	**145**	39	3100	17	192912

Table S4: After filtering out molecules which target EPAS1 and either "TDP1, CFTR, GMNN, PPP1CA" we see no increase in odds for molecules which target only "EPAS1" to produce cytotoxic effect in assay "Luminescence Cell-Based Primary HTS to Identify Inhibitors of Cancer Stem Cells"

Target(Gene/pattern)	P-value FDR adjusted	Odds Ratio	kA	KA	kB	KB
EPAS1	1.00e–300	**21.4**	440	3100	1480	192912
**EPAS1** not **TDP1, CFTR, GMNN, PPP1CA**	0.99	**1.6**	16	2676	629	192061

Data for "HTS Cytotoxicity/Cell viability assay (HPDE-C7K cells)"

Table S5: Top individual targets enriched among molecules producing cytotoxic effect in "HTS Cytotoxicity/Cell viability assay (HPDE-C7K cells)"

#	Target (Gene)	P-value FDR adjusted	Odds Ratio	kA	KA	kB	KB
1	GMNN	1.21e–260	18.7	361	1032	1254	44778
2	TDP1	9.35e–243	9.0	642	1032	6947	44778
3	IDH1	8.53e–211	29.0	234	1032	448	44778
4	JAK2	4.12e–141	25.5	162	1032	324	44778
5	HTR1E	5.86e–124	12.5	200	1032	844	44778
6	TUBB	2.61e–105	20.2	132	1032	323	44778
7	APP	1.50e–95	11.8	157	1032	670	44778
8	HTR1A	8.52e–92	12.1	148	1032	609	44778
9	CFTR	3.03e–87	22.4	104	1032	223	44778
10	CTNNB1	4.08e–87	44.9	82	1032	86	44778

Table S6: Poly-pharmacology of molecules targeting IDH1 in "HTS Cytotoxicity/Cell viability assay (HPDE-C7K cells)"

Target(Gene/ pattern)	P-value FDR adjusted	Odds Ratio	kA	KA	kB	KB
IDH1	8.53e–211	**29.0**	234	1032	448	44778
GMNN	1.21e–260	**18.7**	361	1032	1254	44778
MMP14	2.70e–61	**13.9**	90	1032	306	44778
IDH1 and GMNN	6.38e–143	**49.7**	134	1032	134	44778
IDH1 and MMP14	7.25e–57	**63.3**	50	1032	36	44778
MMP14 and IDH1 and GMNN	1.83e–53	**127**	34	1032	12	44778

Table S7: After filtering out molecules which target IDH1 and either "TDP1,CYP2C9,CHRM4, HTR1E" we see no increase in odds for molecules which target only "IDH1" to produce cytotoxic effect in assay "HTS Cytotoxicity/Cell viability assay (HPDE-C7K cells)"

Target(Gene/pattern)	P-value FDR adjusted	Odds Ratio	kA	KA	kB	KB
IDH1	8.53e-211	**29.0**	234	1032	448	44778
IDH1 not **TDP1, CYP2C9, CHRM4, HTR1E**	0.99	**1.8**	3	801	95	44425
